# Seasonal variations in moderate-to-vigorous physical activity among children and adolescents with visual impairment during different segments of the school day

**DOI:** 10.3389/fpubh.2025.1566561

**Published:** 2025-05-09

**Authors:** Li Dou, Huawei Chen, Yemao Xia, Enjie Lu

**Affiliations:** ^1^Department of Physical Education, Nanjing Forestry University, Nanjing, China; ^2^Department of Physical Education, Nanjing University of Aeronautics and Astronautics, Nanjing, China; ^3^College of Science, Nanjing Forestry University, Nanjing, China; ^4^Department of Physical Education, Nanjing Blind School, Nanjing, China

**Keywords:** seasonal variations, children and adolescents, visual impairment, moderate-to-vigorous physical activity, school day

## Abstract

**Background and objective:**

The seasonal variation in physical activity (PA) among children and adolescents with visual impairment (VI) is a significant public health concern, as they often experience unique barriers to engaging in regular PA. This study examines the seasonal variation in accelerometer-assessed PA among children and adolescents with VI during four distinct segments of the school day: physical education (PE) class, recess, lunchtime, and one-hour club time.

**Participants and methods:**

A total of 63 children and adolescents aged 7 to 17 years from a specialized VI school in eastern China participated in the study. PA was measured using ActiGraph® accelerometers during winter and summer in 2022–2023 academic year. A one-way analysis of variance (ANOVA) was performed to compare the effects of seasonal variation on the time spent on moderate-to-vigorous physical activity (MVPA) by children and adolescents with VI across the four segments of the school day. Additionally, linear mixed models were conducted to estimate seasonal variation in MVPA proportions.

**Results:**

The results revealed that children and adolescents with VI engaged in a higher level of MVPA during winter compared to summer. Across both seasons, primary students exhibited significantly higher cumulative MVPA than secondary students during four school day segments. PE classes were found to constitute the highest percentage of MVPA. Furthermore, the proportion of time spent in MVPA during winter was higher than in summer during recess and lunchtime. Gender differences were also noted, with boys exhibiting higher levels of MVPA than girls during recess and PE time. Additionally, grade-level differences were identified during recess, PE, and lunchtime.

**Conclusion:**

The findings suggest that seasonal factors should be considered when designing physical activity programs for children and adolescents with VI. In particular, more PE classes should be provided and strategies should be adopted to increase MVPA levels during recess and lunchtime in both seasons, which may help children and adolescents with VI to meet the internationally recommended PA standards. Future research should explore the underlying factors influencing seasonal variations in PA among this population and develop targeted interventions to promote MVPA engagement.

## 1 Introduction

Physical activity (PA) is associated with well-documented benefits for physical, social, and mental health (1) such as altering body composition, enhancing cardiovascular function, lowering blood pressure, and alleviating symptoms of depression, anxiety, and poor self-concept (2). Due to its great importance for development and health, PA of children and adolescents has become a key issue in research over the last decade (3). However, the continued decline in PA levels among children and adolescents has caused concerns in many countries, in particular among those with disabilities (4, 5). The World Health Organization (WHO)'s Global Action Plan on Physical Activity 2018–2030 explicitly highlights that promoting PA among people with disabilities is a critical objective for achieving universal health coverage (6).

Participation in PA is crucial for the healthy development of physical fitness and the overall wellbeing of children and adolescents, including those with disabilities (7, 8). PA can provide multiple benefits for children with visual impairments (VI) (9). Unfortunately, Children and adolescents with VI have fewer opportunities to engage in PA compared to their peers without disabilities (10). Studies have reported that children and adolescents with VI are less physically active than those with typical vision (11, 12), and they are known to be at higher risk of an inactive lifestyle, which may lead to a higher prevalence of overweight and obesity compared to typically developing peers (13). To enhance physical fitness and overall health in this group, WHO recommends all children and adolescents achieve at least 60 min of moderate-to-vigorous physical activity (MVPA) daily. However, research indicates that most children and adolescents with VI do not meet this recommendation (14, 15). As a country with a large population, China is confronted with a significant challenge regarding visual disabilities. According to the latest statistics from the China Disabled Persons’ Federation, there are approximately 17 million individuals with visual disabilities in China, among whom around 181,000 are children and adolescents (16). Therefore, increasing the PA levels and decreasing the sedentary time (ST) of youths with visual impairments is important and urgent (17).

MVPA is defined as activity with an intensity equivalent to 3–8.7 metabolic equivalents (18). Children and adolescents who participate in MVPA are more likely to maintain these healthy habits into adulthood (19). MVPA is an essential component of health interventions. Several studies have demonstrated that children and adolescents with VI tend to spend less time in MVPA and more time in sedentary behavior (20–22), because various environmental barriers may prevent children and adolescents with disabilities from participating in adequate levels of MVPA (23). Recently, researchers have highlighted that surrounding environmental factors, for example, seasonal variation, which can play an important role in influencing PA and sedentary time of children (24). Seasonal factors including weather, temperature, and daylight hours, can significantly affecting PA levels of children and adolescents, particularly among children and adolescents with disabilities. For example, extreme temperatures, strong winds, rain, and snow are all likely to reduce children’s PA levels. Some studies have reported the lowest PA levels in winter (25), while others have found the lowest PA intensity in summer and the highest in winter (26, 27). These inconsistent findings highlight the need for a comprehensive study of seasonal variations in PA among children and adolescents with VI. Because seasonal meteorological factors cannot be avoided, identifying the effects of seasonal characteristics on PA and sedentary behavior is crucial for developing public health interventions aimed at promoting PA and reducing sedentary time in schools (28). Unfortunately, limited studies have objectively measured PA among children and adolescents with VI. Specifically, there is a lack of data on seasonal variation in MVPA among children and adolescents with VI in China.

Socioecological models have clearly indicated that the PA of children and adolescents with VI is greatly affected by multiple factors, including individual, family, community, and cultural factors (29). Among these, schools are the major settings for children and adolescents with VI to accumulate their MVPA time, as they provide opportunities such as physical education (PE) class, lunchtime, recess, and extracurricular activities (30). A further understanding of how children and adolescents with VI accumulate PA during the entire school day is essential for developing effective school-based PA promotion programs. Previous studies have primarily focused on assessing PA levels of children and adolescents with disabilities during different segments of the school day (27, 31, 32). However, limited research has specifically examined the PA levels of children and adolescents with VI. Additionally, some demographic characteristics, such as gender and age, have been widely reported to affect the segmented PA participation of children and adolescents without disabilities (33, 34). Whether these factors affect the segmented PA of children and adolescents with VI remains unknown. Further studies are needed to explore these factors of PA among children and adolescents with VI. Therefore, this present study examines the seasonal variation in accelerometer-assessed PA among children and adolescents with VI during four distinct segments of the school day, which is important for schools and education departments to create and implement effective comprehensive school PA promotion programs.

## 2 Participants and methods

### 2.1 Participants

Participants were recruited in 2022–2023 from a VI school in Jiangsu Province in east China. The school is renowned for its comprehensive education system for children and adolescents with VI, integrating preschool education (ages3–6 years), primary education (ages7–12 years), secondary education (ages 13 years and above), vocational training, and social skills development. Participants were invited to participate in this study if they were aged between 7 to 17 years who had low vision or blindness, and were free from other medical, intellectual, or physical disabilities. Briefly, participants in grades 1–12 were invited to participate, and parental written consent and child assent were provided for 70 participants. The school provided the background information on the participants, including the gender, age, grade and detailed schedules.

Data collection was conducted during winter (November–December 2022, monthly mean temperature 7–13°C, relative humidity 67%–73%) and summer (June–July 2023, monthly mean temperature 26–32°C, relative humidity 76%–81%). During the study, some children and adolescents withdrew due to illness, and while others failed to adhere to protocol of wearing the accelerator. Consequently, the final analytic sample consisted of 63 participants. Table 1 presents the detailed characteristics of the study sample.

**Table 1:** The demographic characteristics of the participating students with VI (total *N* = 63 with valid data during both winter and summer).

Demographic	Full sample (*N* = 63)	Boys (*N* = 38)	Girls (*N* = 25)
	Mean (SD)	Mean (SD)	Mean (SD)
Age	11.78 (2.55)	11.97 (2.72)	11.48 (2.78)
Height (cm)	156.77 (9.21)	157.38 (10.28)	155.84 (7.41)
Weight (kg)	51.68 (7.43)	51.28 (7.49)	52.28 (7.44)
BMI (kg/m^2^)	20.97 (1.86)	20.64 (1.58)	20.97 (1.85)

Grade	*N* (%)		
Grade 1–6	34 (53.97)	19 (55.88)	15 (44.12)
Grade 7–12	29 (46.03)	19 (65.52)	10 (34.48)

Data are shown in mean (SD). BMI, body mass index.

### 2.2 Physical activity measures and procedures

The school schedules were used to identify the specific time periods for PE class, recess, lunchtime, and club time sessions (Table 2). The schedule includes three 40-min PE classes per week for primary students and two 40-min classes PE lessons per week for secondary students. During break time, the majority of children and adolescents with VI tended to remain in classrooms or engage in social interactions with their peers. In contrast, a smaller proportion of them participated in unstructured PA, such as spontaneous rope skipping, sliding, climbing, ball bouncing, and informal sports games. During the one-hour club time periods (16:30 to 17:30), children and adolescents with VI can participate in various sports training and group activities organized by the school, such as rope skipping practice, chorus, and goalball.

**Table 2:** Time segments during the school day.

Segment	Time	Min	Main contents
Recess	08:10–08:40	30	Sports break, morning exercise
	09:20–09:30	10	Between the first and the second classes
	10:10–10:20	10	Between the second and third classes
	11:00–11:10	10	Between the third and fourth classes
	13:50–14:10	20	Long break, special sports activities
	14:50–15:00	10	Between the sixth and seventh classes
	15:40–15:50	10	Between the seventh and eighth classes
	Total	100	
Lunchtime	11:50–13:10	80	Lunch and break
One-hour club time	16:30–17:30	60	Jump rope or other training exercises

For five consecutive school days in each season, participants were required to wear an ActiGraph^®^ accelerometer (wGT3X-BT model, ActiGraph^®^, Pensacola, FL, USA), a widely recognized instrument for the objective measurement of the PA and sedentary time in children and adolescents, including those with disabilities. Each accelerometer was assigned a unique identification number and was allocated to the participants with the corresponding identification number. The participants were instructed to start wearing the device, which was appropriately attached above the student’s waist via an elastic belt.

The cut-off points (≥2,296 counts per minute) of the PA of children and adolescents advocated by Evenson et al. were used classify MVPA levels (35), and they have been validated for use in children and adolescents with disabilities (3, 21, 27). Accelerometer data were recorded in units of counts/15 s. For a valid school day, the minimum required daily wear time on campus was 480 min.

To ensure data accuracy, one author and trained research assistants visited the school each morning to check the functionality of the accelerometers and to record the weather conditions during the study period, supplementing weather forecast information. For each day, MVPA was calculated for the entire school day and separately for each of the four different school-day segments identified in Table 2. Assessment days were consecutive, and at least one day of each season included a PE lesson. Participants were asked to record the time they removed the device and reasons for doing so. The time for which all participants could be absent from their devices was limited to less than 10% of the day. The participants were asked to follow their normal daily routines during the monitoring period. After the test, the raw ActiGraph data were processed using the ActiLife Lifestyle Monitoring System (software version 6.13.3; ActiGraph, Pensacola, FL, USA).

### 2.3 Statistical analysis

Descriptive statistics were initially calculated for all measured variables, with results presented as mean ± standard deviation (SD). The total accelerometer wear time was determined, and the duration as well as proportion of time spent in MVPA were calculated for four distinct segments of the school day. To assess the normality of the data, the Shapiro–Wilk test was conducted. If the data were normally distributed, a one-way analysis of variance (ANOVA) was performed to compare the time spent in MVPA during the entire school day and across four distinct segments, with season, gender, and grade included as factors. Additionally, linear mixed models were used to evaluate the impact of season, gender, and grade on MVPA proportions with participants as random effects. In these models, MVPA% during recess, PE lessons, lunchtime, and club time were each separately treated as dependent variable. The models were adjusted for potential confounding factors, including disability level, BMI and total wearing time. If the data were not normally distributed, the Friedman test was used to increase the robustness of the results. All statistical analyses were conducted using SPSS^®^ software version 25.0 (IBM Corp, Armonk, USA), and a significance level of 0.05 was considered to indicate statistical significance.

## 3 Results

### 3.1 MVPA% overall and during the four segments of the school day in both seasons

The participants wore the accelerometers at school from 08:00 to 17:30, thereby constituting a total of 570 min per day during winter and summer. On average, participants engaged in 8.11% of their time to MVPA during school days in winter. In contrast, the proportion of MVPA during the school day was 7.63% in summer. The primary summary of the MVPA% data collected in the four different segments of the school day during winter and summer for all students are presented in Table 3. Regardless of the season, the proportion of MVPA during PE classes was the highest (29.08 and 27.81%) in winter and summer respectively, followed by that during the one-hour club time (18.08 and 19.05%), and the proportion of MVPA time during recess was the lowest (12.33 and 11.07%).

**Table 3:** MVPA% overall and during the four segments of the school day in both seasons.

School day segment	MVPA (%) in Winter	MVPA (%) in Summer
	Mean (SD)	Mean (SD)
Recess	12.33 (0.03)	11.07 (0.02)
PE	29.08 (0.07)	27.81 (0.06)
Lunchtime	14.28 (0.03)	12.32 (0.04)
Club time	18.08 (0.04)	19.05 (0.05)
Total	8.11 (1.22)	7.63 (1.09)

Data are shown in mean (SD). PE, physical education; MVPA, moderate-to-vigorous physical activity; MVPA %, percentage of MVPA time.

### 3.2 Seasonal and demographic comparisons of MVPA time

**Figure 1:**
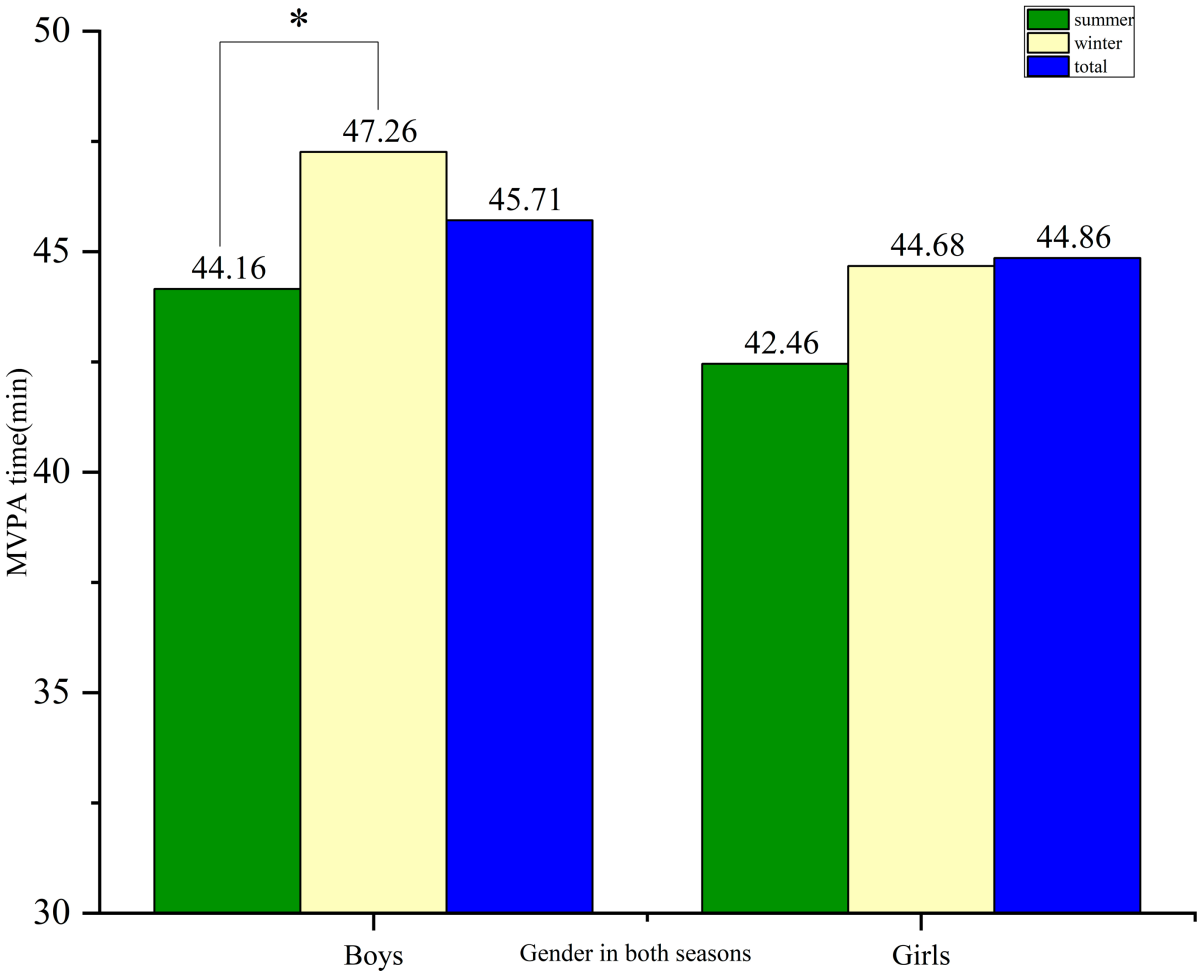
The difference in the overall time spent in MVPA between boys and girls in both seasons. *p < 0.05, ** *p* < 0.01.

The boys accumulated more MVPA time than girls across all four segments of the school day, regardless of season (Figure 1). However, this difference was not statistically significant. Among boys, cumulative MVPA time was significantly higher in winter compared to summer (47.26 vs.44.16 min, *p* = 0.04), whereas no significant seasonal difference was observed among girls (44.68 vs.42.46 min, *p* = 0.08).

**Figure 2:**
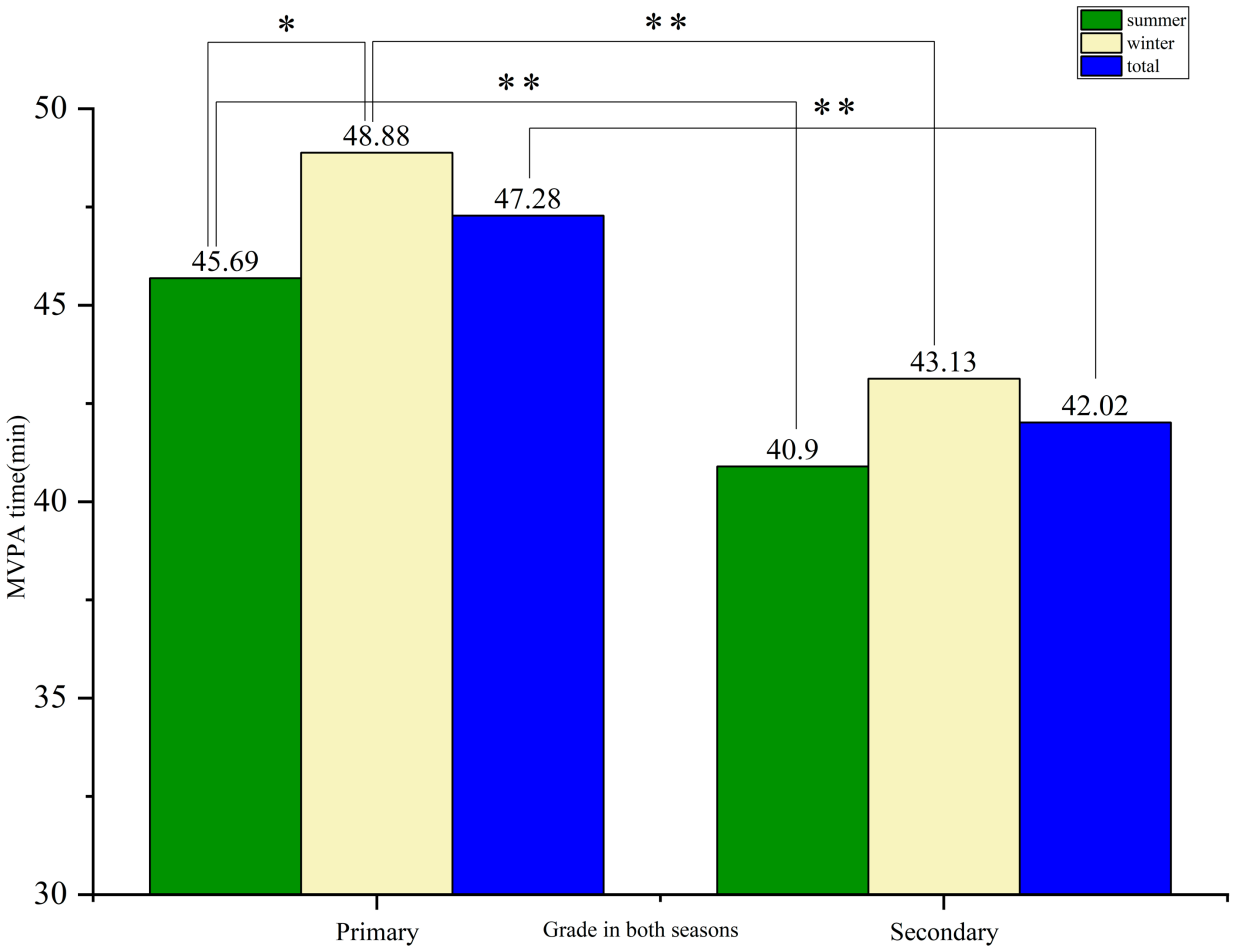
The difference in the overall time spent in MVPA between primary and secondary school students in both seasons. *p < 0.05, ** *p* < 0.01.

The cumulative MVPA time of primary school students in the four segments of the school day were significantly higher than that of the secondary school students in both seasons (winter 48.88 vs. 43.13, *p* = 0.002; summer 45.69 vs. 40.90*, p* = 0.001) (Figure 2). A seasonal difference was found in the cumulative MVPA time of primary school students (48.88 vs. 45.69, *p* = 0.02), but no seasonal difference was found for secondary school students (43.13 vs. 40.90, *p* = 0.137).

### 3.3 Seasonal variation in MVPA% during recess, PE lessons, lunchtime, and club time

Results from the linear mixed models analysis (Table 4) indicated that there was a seasonal difference in MVPA% during recess (*β* = −0.01, 95%CI = −0.02,0.00, *p* = 0.003) and lunchtime (*β* = −0.02, 95%CI = −0.03,0.00, *p* = 0.002), whereas there was no seasonal difference during PE class and one-hour club time. Boys were more physically active than girls during both recess time (*β* = 0.01, 95%CI = 0.00,0.02, *p* = 0.02) and PE Class (*β* = 0.04, 95%CI = 0.01,0.06, *p* = 0.002). Furthermore, grade-level differences in MVPA% were identified during recess (*β* = 0.01, 95%CI = 0.00,0.02, *p* = 0.01), PE class (*β* = 0.04, 95%CI = 0.01,0.06, *p* = 0.003), and lunchtime (*β* = 0.03, 95%CI = 0.01,0.04, *p* = 0.001). Primary students exhibited higher MVPA levels compared to secondary students during these segments, suggesting that younger students with VI were more active. No significant grade-level differences were observed during club time.

**Table 4:** Seasonal variation in MVPA% during recess, PE lessons, lunchtime, and club time.

Variable	Recess MVPA %			PE MVPA%			Lunchtime MVPA%			Club time MVPA%		
	*P* value	95% CI	Coefficients	*P* value	95% CI	Coefficients	*P* value	95% CI	Coefficients	*P* value	95% CI	Coefficients
Season (reference: winter)	**0.00**	**−0.02,0.00**	**−0.01**	0.28	−0.04,0.01	−0.01	**0.00**	**−0.03,0.00**	**−0.02**	0.21	0.00,0.02	0.01
Gender (reference: girls)	**0.02**	**0.00,0.02**	**0.01**	**0.00**	**0.01,0.06**	**0.04**	0.07	−0.00,0.02	0.01	0.09	−0.03,0.00	−0.01
Grade (reference: secondary)	**0.01**	**0.00,0.02**	**0.01**	**0.00**	**0.01,0.06**	**0.04**	**0.00**	**0.01,0.04**	**0.03**	0.06	0.0,0.02	0.01

Findings were derived from linear mixed models with students as random effects, and MVPA% during recess, PE lessons, lunchtime, and club time was each separately treated as dependent variable in the models which were adjusted for disability level, BMI and total wearing time.

MVPA%, MVPA percentage of wear time. Statistically significant results are in bold.

## 4 Discussion

To the best of the authors’ knowledge, limited research related to the PA of individuals with VI has been conducted in China. This is one of few investigations to examine the seasonal variation in the accelerometer-assessed MVPA among children and adolescents with VI. This study also described and compared the duration of MVPA of children and adolescents with VI during different segments of the school day in Chinese school in winter and summer. The findings provide valuable insights into how PE and recess can influence the MVPA of children with VI in special school settings.

Our study identified a distinct seasonal variation in the MVPA levels of children and adolescents with VI, with objectively measured MVPA being significantly lower during summer compared to winter across the four segments of the school day. This finding is consistent with previous research on typically developing children, which also reported reduced MVPA levels during summer (36). Similarly, a study of special schools in Hong Kong that included children and adolescents with VI demonstrated that children with various disabilities are generally more active at school in winter than in summer (27). One reason for this may be related to the local hot and humid summer climate, as humid and sultry weather leads to the reduction of MVPA for children with VI. The school considered in the present study is located in the middle and lower reaches of the Yangtze River, and experiences a typical hot and humid summer climate in June.

It appears that hot and humid weather has a greater influence on the MVPA of children with VI, as high temperatures and humidity can exacerbate physiological strain and discomfort, making physical activities less appealing. This explanation is supported by the findings of Wallenber et al. (37), who revealed that hot weather conditions could have negative impacts on the thermal comfort and physical activity of vulnerable groups such as children. Another possible reason is that children and adolescents with VI wear more clothing to make them feel safer when engaging in PA during winter, because additional layers can offer better protection from falls or collisions due to limited vision from a logical perspective. Regardless of the season, PE class was identified as the highest percentage of MVPA, followed by one-hour club time, while recess time constituted the lowest percentage of MVPA. This finding is not consistent with the results of some previous studies, which found that children engaged in a greater proportion of MVPA during recess than during PE lessons (38). This discrepancy may be attributed to variations in the organization and structure of PE and recess activities across different countries and educational settings. However, a recent survey of schools for the blind in western China further supported that structured PE classes had a higher percentage of MVPA time than recess, lunchtime, and after-school periods during the school day (17). In that study, the participants with VI were found to engage in significantly more MVPA time on PE days than on non-PE days. During PE time, children and adolescents were closely supervised by teachers who provided guidance and skills on how to engage in exercises safely, which probably made children and adolescents feel comfortable and thereby increased their time spent in MVPA. These findings underline the critical role of teacher in structured PE classes in promoting MVPA among children and adolescents with VI. Given the importance of PE classes as a primary opportunity for MVPA, targeted interventions during structured PE classes are highly recommended, and well-trained adapted PE teachers are essentially indispensable. Therefore, it is both necessary and crucial to add PE classes in schools for children and adolescents with VI. Future efforts should focus on increasing the quantity and quality of PE classes to enhance their MVPA levels.

The present study reveal no significant seasonal differences in the percentage of MVPA during PE classes. This finding may be attributed to the structured nature of adapted physical education programs in primary and secondary schools. These programs were characterized by a formalized teaching syllabus and prescribed content, as well as specific intensity requirements for in-class exercises. The consistency in MVPA% across seasons was a result of these structured lesson elements, which ensured that PA levels remain stable regardless of seasonal variations. In contrast, seasonal differences were found in MVPA% during recess and lunchtime. Specifically, we observed the higher MVPA levels during lunchtime and recess in winter compared to summer. This may be attributed to the low temperature in winter, and children and adolescents may engage in more PA to keep warm. Conversely, the temperature during lunchtime is higher in summer, and children and adolescents are more willing to do light activities or rest quietly, which may lead to reduced MVPA%. In addition, activities during lunchtime and class breaks are independent activities, making them highly susceptible to external influences such as weather conditions and personal preferences. Although there are 100 min of school break time and 80 min of school lunchtime, the accumulated MVPA during these periods is relatively low, particularly in summer. Interestingly, a previous study reported that children with VI accumulated more MVPA during recess, and this resulted from teachers in the VI school having organized many orientation and mobility activities focused on developing the functional skills and gross motor tasks of children and adolescents, including walking (39). These findings suggest that programs should be actively developed to promote PA during breaks, and more opportunities should be provided for children and adolescents with VI to improve their MVPA.

To date, limited studies have examined seasonal variation in PA of children and adolescents across different age subgroups. Overall, the PA levels of children and adolescents generally decline with age (25, 40, 41). Consistent with this trend, in the present study found MVPA levels decline as children and adolescents progressed from primary to secondary school, with primary school students exhibiting significantly higher MVPA% compared to secondary school students during recess, PE classes and lunchtime. In contrast, older adolescents may be less influenced by seasonal changes, possibly due to greater independence and more varied activity preferences. In an addition, the transition to secondary school is often accompanied by increased academic demands and longer school hours in China, which can reduce the time available for MVPA. In both seasons, gender difference was observed in the MVPA% during recess and PE class, and this finding is similar to a previous systematic review (42), which found that boys spent significantly more time in MVPA compared to girls. These findings highlight the need for targeted interventions to promote MVPA among girls, particularly in school settings. Additionally, the results demonstrate that the PA of primary school students, especially boys, are more easily influenced by seasonal factors (such as temperature and humidity). This highlights the necessity for schools to incorporate seasonal considerations into their PA arrangements, especially during recess and lunchtime. Seasonal sports can be appropriately arranged to improve the MVPA level of primary school students in summer. Previous studies have reported students with VI can achieve comparable PA levels to their sighted peers when provided with equal opportunities for regular physical activity (43, 44). Therefore, VI schools should not only provide a curriculum with high expectations but also create opportunities and incentives to engage in PA via physical education and seasonal extracurricular sports. This intervention has the potential not only to enhance physical health outcomes but also to improve holistic well-being among children and adolescents with VI.

## 5 Conclusion

The present research on the seasonal variation of MVPA among children and adolescents with VI in China revealed that the levels of MVPA were higher during winter than in summer, particularly during recess and lunchtime. The MVPA of boys and primary school students was found to be more susceptible to seasonal influences. The seasonality of MVPA among children and adolescents with VI can be influenced by relevant background factors, such as gender, time segments, PE curriculum, weather, and temperature. Collectively, these factors offer insights for developing targeted interventions to promote MVPA among children and adolescents with VI across different seasons.

## 6 Strengths and limitations

The strengths of the study included the use of objective measures to assess PA. Linear mixed models were employed to account for addressing potential within-group correlations among disability level, gender, grade, BMI and total wearing time during different school segments. However, some limitations should be noted. Firstly, assessments were completed only during two seasons, and future studies should include multiple seasons to provide a more comprehensive understanding. Secondly, the children and adolescents who volunteered for this study may have been those who were more physically active than the nonparticipating counterparts. Thirdly, participants were recruited from only one school in east China, and it is possible that these findings are not representative of schools located throughout China. Further studies are needed from other areas of China to identify potential variable differences due to geographical, climatic, or cultural disparities. Furthermore, more research investigating the associations between contextual factors and PA in children and adolescents with VI is needed. Future studies should determine how such variables may influence activity levels in different seasons, thereby providing valuable insights into the development of targeted, season-specific interventions to promote MVPA among children and adolescents with VI.

## Data Availability

The raw data supporting the conclusions of this article will be made available by the authors, without undue reservation.
